# The Common Accidents of Every-Day Life

**Published:** 1888-01-14

**Authors:** Robert A. P. Forrester


### The Common Accidents of Every-day Life.

By Robert A. P. Forrester, B.A.Dub., M.B. and C.M.Edin.

**Childhood: Greenstick Fracture.**

This fracture sometimes occurs in young children before the bone is thoroughly ossified. A young child, aged between three and four years, had fallen from a chair upon the floor, a distance of only two feet. When I saw it the right fore-arm was considerably bent, and on examination I did not find the usual symptoms of fracture, and concluded it was a "green-stick." Great difficulty was experienced in moulding the limb into its proper form. I therefore put the child under chloroform, and, making use of considerable force, broke the bones completely, and put the arm up in splints. Great care should be exercised in applying splints to the forearm, and especially must we see that the bandages are not put on too tightly. If this is unfortunately done, there is danger of gangrene coming on from arrest of the circulation. Numerous instances have been recorded of this unfortunate accident, and it is not always the fault of the surgeon, for it may be due either to the carelessness of the parents or the patient himself. It is easy to see how gangrene may occur, for both the principal arteries in the forearm are superficial, and therefore specially liable to the influence of undue pressure.

I give one instance merely as an illustration. A woman, aged about fifty, fell upon her left hand, causing a compound fracture of the radius and ulna about three inches above the wrist-joint. She was removed to a dispensary, where the surgeon put the arm up in splints, applying the bandages "snugly." Two days afterwards she was removed to one of the hospitals in the town where she resided, and was seen by one of the surgeons. The back of her hand and most of the forearm were gangrenous, evidently caused by the bandages. A few days after, she died from secondary hæmorrhage, before the house surgeon could reach her.

Splints, therefore, must be carefully applied. They should be *broader* than the arm; the back splint should reach from beyond the elbow to the tip of the fingers, and another short splint placed in front of the limb, reaching from the elbow to the roots of the fingers. They should be of the same breadth. Gooch splinting will do very well, or two thin, firm, wooden splints may be made. After being properly padded with tow or cotton wool, they must be applied whilst the arm is held at right angles to the elbow, and in a position midway between pronation and supination. If you lay your forearm flat on the table, with the palm of the hand looking upwards, the arm is said to be "supinated"; now, if you turn the hand round without removing the arm from the table, and cause the back of the hand to look upwards, you will have the arm "pronated." One splint—the longer of the two—must be placed on the dorsal surface, and the other on the palmar aspect of the arm, extension and counter-extension being of course maintained all the time, and then the whole must be secured with a bandage. After this a sling is adjusted. Some surgeons used to apply a roller or bandage to the skin before putting on the splints, but this practice does no good, and may do a great deal of harm.

The arm should be examined once every twenty-four hours during the first ten days at least, to see that everything is in position, that the bandages are neither too tight nor too loose. Some surgeons, indeed, use no bandages at all, but merely fasten the splints with straps. It is a good plan in fracture of both bones to put a pad on the front of the arm; this forces the muscles down into the interosseous space—*i.e.*, the hollow between the bones—and prevents displacement of the bones in that direction.

**Stings from Insects.**

Stings from bees, wasps, and other insects are not uncommon. A dozen wounds at once from this source may prove fatal.

When a boy, I more than once waged war upon wasps. I remember "striking oil" in the shape of a nest upon one occasion, and after gravely consulting with a friend upon the best method of attack, we decided upon blowing it up with gunpowder. Our preparations were elaborate; our faces and necks were well protected with muslin; our hands with gloves. Into the entrance of the nest we poured a considerable quantity of gunpowder, and laid a train for some distance from it. A match was applied, and the result was eminently satisfactory to us. The nest was demolished, but we did not escape "scot-free," for the wasps were irritated pretty considerably. The reasoning faculty seemed fairly developed in these particular ones. They saw the effect, and arrived instantly as to the cause. Had we been attentive to the teaching of Arnold's First Latin Book, in one part of which the author advises his young friends, "Ne irrita vespas" ("Do not irritate wasps"), we might not have suffered the consequences of our disobedience to that most excellent maxim. The wasps came upon us in myriads, and now we found the muslin stand us in good stead. I was the victim to a considerable extent, for I was stung in four places.

A medical gentleman of my acquaintance was stung on the tongue by a bee or a wasp in rather a curious manner. He was sitting at lunch one day talking to a friend, and mechanically picked up what he thought to be a piece of crust and put it into his mouth. It proved, however, to be an insect of the bee or wasp variety, which naturally resented the liberty taken with it. The tongue began to swell, and suffocation might have occured had it not subsided. Every preparation was made for performing tracheotomy, but fortunately this was not necessary.

What is a sting? It is an abnormal sensation caused by the introduction into the system of an irritant or poisonous fluid, and may be said to be a combination of pain and itchiness. The fluid in question is thought to be of the nature of formic acid.

The introduction of the fluid is followed very shortly after its reception by redness and swelling. If the mucous membrane, as of the mouth, be the affected part, the swelling is very great; the tongue cannot be protruded, and swallowing is impossible. Deaths from the stings of scorpions, bees, and wasps have been recorded. In the majority of such cases, faintness, severe prostration, and pallor are the prominent symptoms, and death has been known to take place in a few hours.

What is the best method to pursue in such accidents? (1) Local treatment. Liquor ammoniæ, or a solution of some other alkali, such as bicarbonate of soda or potash, if applied at once, has been found efficacious. Carbolic oil (1-20) is also good. Ipecacuanha powder is said to be beneficial. First of all, however, if you can see the sting, extract it immediately, and then apply one or other of the above remedies. To overcome the constitutional effects, such as faintness, &c., give freely of brandy-and-water, ether, or ammonia. These are imperatively necessary, for life depends upon counteracting the extreme depression of the heart. Before leaving the subject of stings, I may mention that in nettles the stinging fluid is believed to be either oxalic or acetic acid. It is thought to be stored in the hair, which terminates in a bent knob. This knob is broken off when brought into contact with the skin, and the fluid escapes into it. Stinging organs are also found in other animals besides bees, wasps, scorpions, etc. The most familiar specimen of a stinging animal is a jellyfish, or sea-nettle. The organs consist of little cells containing an acid fluid, and are prolonged into a thread which has many barbs upon it. The thread is usually coiled in a spinal manner within the cell, from which it is everted upon contact, conveying the fluid into the surface it penetrates.

*(To be continued.)*

